# Hybrid frequency-domain CFAR detectors for cognitive radio interference resilience and the dual-use security paradox

**DOI:** 10.1038/s41598-026-63489-w

**Published:** 2026-07-30

**Authors:** Mohamed Salah Shams, Ahmed A. Abouelfadl, Ahmed Mansour, Mohamed Samir Abdel Latif Soliman

**Affiliations:** 1 Electrical Engineering Branch, Military Technical College, Cairo, Egypt; 2 Electronic Warfare Department, Military Technical College, Cairo, Egypt

**Keywords:** Cognitive radio, Spectrum sensing, CFAR detection, Hybrid architectures, Jamming countermeasures, Algorithm-aware jamming, Engineering, Mathematics and computing

## Abstract

Cognitive radio networks (CRNs) enable secondary users (SUs) to opportunistically access underutilized licensed spectrum while protecting primary users (PUs). Robust spectrum sensing under heterogeneous interference and heavy jamming remains challenging: conventional approaches either require extensive prior knowledge or degrade significantly under interference, while basic constant false-alarm rate (CFAR) variants remain vulnerable to structured jamming that contaminates only part of the reference window, causing fixed-rule estimators to either include contaminated samples or discard clean ones. Hybrid CFAR architectures address this by first analyzing reference-window contamination patterns and then adaptively selecting or combining estimation strategies. We adapt eleven such hybrid architectures—originally developed in the radar literature—to frequency-domain spectrum sensing in contested CRNs, and evaluate them across four families (adaptive algorithm selection, order-statistic fusion, intelligent censoring, and weighted processing) against classical CR-CFAR baselines via Monte Carlo simulations using APCO Project 25 as the PU waveform and an OFDMA-based SU, under barrage and swept-FM jamming. Results show that First-Order Difference CFAR achieves the best detection performance through global sorting and statistical jump detection ($P_{\mathrm{d}} \approx 1$ at SNR *= 3* dB under 50% sweep coverage), followed by Smallest-Of and Order-Statistic Smallest-Of variants. However, this resilience creates a dual-use security paradox: unauthorized devices employing these techniques can resist administrative spectrum control. We address this through an algorithm-aware comb-sweep countermeasure that exploits CFAR reference-window dependencies, inducing near-unity false alarms on vacant channels within a 4 dB JSR denial plateau (*-5* to *-1* dB) while preserving PU detection, demonstrating that algorithm-aware enforcement can restore spectrum governance in contested environments.

## Introduction

The proliferation of wireless devices and services has intensified spectrum scarcity, even though substantial portions of the licensed spectrum remain underutilized across time, location, and frequency. Cognitive radio (CR) networks address this inefficiency by enabling SUs to opportunistically access licensed bands when PUs are absent^1^. This dynamic access paradigm hinges on reliable spectrum sensing: SUs must detect weak PU activity and vacate channels promptly to prevent harmful interference^2^. However, practical sensing is challenged by nonstationary interference and intentional jamming, which bias sensing statistics and destabilize false-alarm behavior^3,4^. While noise uncertainty (NU) affects fixed-threshold energy detection, this paper emphasizes structured jamming that contaminates local background estimates.

Before presenting the CFAR-based sensing framework, it is useful to place it within the broader landscape of spectrum sensing techniques. Energy detection (ED) is the most widely adopted approach in CR systems because it is blind, i.e., requires no prior knowledge of the PU waveform^4^. Despite its simplicity, conventional ED relies on fixed thresholds, making performance highly sensitive to NU and interference variations. Matched-filter detection is optimal when the PU waveform is perfectly known^2^, but this requirement is rarely satisfied in opportunistic access settings where PU signaling may be unknown or time-varying. Cyclostationary feature detection exploits periodic modulation structure to distinguish PU signals from noise^5^; it is robust to NU and can mitigate the “SNR wall”, but requires higher computational complexity and longer sensing times^4^. Eigenvalue-based detection leverages the sample covariance matrix structure^6^, operating without a fixed noise reference, but incurs substantial cost due to covariance estimation and eigenvalue decomposition^7^. These complexity—robustness trade-offs motivate adaptive-threshold approaches that maintain low implementation cost while improving resilience to interference^8^.

CFAR detection, originally developed for radar^9^, maintains a prescribed $P_\mathrm{fa}$ by estimating the local interference level and adapting the threshold accordingly. While classical radar CFAR operates over time-indexed range cells^10^, the principle transfers to communication sensing by forming local reference windows over time samples and/or frequency bins^10^. CFAR algorithms can be grouped into two families: (a) mean-level estimators, including cell-averaging (CA-CFAR), greatest-of (GO-CFAR), and smallest-of (SO-CFAR) schemes^11^; and (b) robust rank- and censoring-based estimators, such as order-statistic CFAR (OS-CFAR) and censored CFAR^12,13^. However, under heavy or structured interference, even these classical variants can suffer when reference cells are contaminated, motivating hybrid CFAR architectures that combine complementary estimators or incorporate explicit contamination-mitigation mechanisms.

Recent work^14^ evaluated five classical CFAR variants adapted to frequency-domain spectrum sensing under diverse jamming conditions, finding that SO-CFAR and Censored-CFAR provided the best performance among conventional approaches. However, that analysis also revealed a fundamental security challenge: the same adaptive sensing that helps authorized users can also help unauthorized transmitters resist spectrum enforcement. This concern is not theoretical;operational systems increasingly face intelligent jamming^3^ and adversarial emitters that mimic legitimate signals to evade detection^15^. These threats indicate that robust sensing techniques must be paired with administrative control mechanisms to maintain spectrum governance^16^.

Hybrid CFAR architectures have been explored in radar^11,12^ and are emerging in communication sensing^10,17^, with recent advances using censoring^13^, order statistics^12^, and machine learning^18^. However, comprehensive evaluation of hybrid CFARs against the classical baselines established in^14^—particularly under algorithm-aware jamming—has not been reported.

Building on these findings^14^, this paper extends the analysis in two ways. First, we evaluate eleven hybrid CFAR architectures that use advanced processing techniques—including adaptive algorithm selection, order-statistic fusion, intelligent censoring, and weighted averaging—to determine if they outperform the SO-CFAR and Censored-CFAR baselines under extreme interference. Second, we develop an improved countermeasure tailored to how hybrid CFARs process reference windows. Our comb-sweep jamming strategy exploits the statistical dependencies in hybrid CFAR thresholding to create constant false alarms at unauthorized receivers (denying them spectrum access) while protecting legitimate PU transmissions. Unlike wideband jamming, this approach achieves enforcement goals with much less jamming power^19^.

The primary contributions are:

1. Adaptation and systematic performance evaluation of eleven hybrid CFAR architectures—originally developed in the radar literature^13,20–28^—to a cognitive radio frequency-domain sensing context under diverse structured jamming. This extends beyond existing radar-domain hybrid CFAR studies, which do not consider communication-sensing jamming, and beyond our own classical CR-CFAR work^14^, which evaluated only five non-hybrid variants, by providing the first comparison of hybrid estimators against classical baselines under cognitive radio jamming conditions, including algorithm-aware counter-access threats.
2. Design and evaluation of an algorithm-aware jamming countermeasure that denies unauthorized spectrum access while preserving PU protection.

The remainder of this paper is organized as follows: “Background and related work” section provides background on cognitive radio paradigms and spectrum sensing techniques, and introduces CFAR concepts and jamming challenges. “System model and frequency-domain CFAR framework” section describes the system model and frequency-domain CFAR sensing framework, including the signal model and CFAR sliding window process. “Resilience of hybrid frequency-domain CFAR under heterogeneous interference and security-oriented threats” section presents performance results for CFAR detectors under representative jamming scenarios (barrage and sweeping), along with complexity analysis for the hybrid algorithms. “Administrator-controlled counter-access via comb-sweep jamming” section proposes an algorithm-aware comb-sweep jamming countermeasure and evaluates its effectiveness in denying spectrum access to CFAR-based SUs while preserving PU communications. “Conclusion and future work” section concludes the paper and discusses future research directions.

## Background and related work

This section provides the background needed to motivate and interpret our proposed framework. We review core cognitive radio (CR) paradigms, summarize spectrum sensing approaches, and highlight practical challenges arising from NU and intentional interference/jamming. We focus on the interweave (opportunistic access) paradigm and introduce the signal modeling elements required for the frequency-domain CFAR formulations developed in the subsequent sections.

### Cognitive radio paradigms

CR networks are commonly categorized into three paradigms: underlay, overlay, and interweave^1^. Each paradigm specifies different coexistence rules that govern how SUs may access spectrum allocated to PUs. In the underlay paradigm, SUs transmit concurrently with PUs subject to an interference constraint at the primary receiver, typically enforced via strict power control and/or wideband signaling^29^. Ultra-wideband (UWB) is a representative example, where very low power spectral density enables coexistence across wide frequency ranges. While underlay access can be continuous, it relies on accurate channel knowledge and robust power control to satisfy interference constraints^30^. In the overlay paradigm, SUs exploit prior knowledge of the PU transmission and may assist the primary link (e.g., via cooperative relaying or coding) while communicating concurrently^31^. Although overlay can improve overall spectral efficiency, it typically requires extensive coordination and information sharing, which introduces additional implementation complexity and expands the threat surface in adversarial settings^32^. The interweave paradigm, also known as opportunistic spectrum access, is the central focus of this paper. It is motivated by the observation that portions of licensed spectrum may be idle at particular times, locations, or frequencies, creating exploitable “spectrum holes” (or “white spaces”)^1^. A prominent real-world example is TV white-space operation, where devices opportunistically use unoccupied broadcast channels subject to regulatory constraints. In the interweave model, an SU must first sense the channel to infer PU absence; only then may it transmit, potentially at higher power than underlay operation. Consequently, sensing accuracy and latency are critical: reliable detection protects PUs from harmful interference while maximizing SU access opportunities^33^.

### Spectrum sensing types

Spectrum sensing in cognitive radio networks is formulated as a binary hypothesis test^34^. The received baseband signal $y_s(t)$ at the SU satisfies

1 $$\begin{aligned} {\left\{ \begin{array}{ll} \mathcal {H}_0: y_s(t) = n(t) + j(t), & \text {PU absent}, \\ \mathcal {H}_1: y_s(t) = s_p(t) + n(t) + j(t), & \text {PU present}, \end{array}\right. } \end{aligned}$$

where $s_p(t)$ is the PU signal, *n*(*t*) is thermal noise, and *j*(*t*) is interference or jamming. In this formulation, *n*(*t*) is modeled as a random process with i.i.d. samples $n[n]\sim \mathcal{C}\mathcal{N}(0,\sigma ^2)$. The interference term *j*(*t*) is treated as a deterministic structured waveform whose power level parameterizes the jammer-to-signal ratio (JSR). The PU signal $s_p(t)$ is likewise treated as deterministic with known bandwidth, consistent with the APCO P25 waveform used in simulations; its average power varies across Monte Carlo trials via the SNR sweep.

Irrespective of the sensing strategy, the receiver computes a test statistic $T(y_s)$ from the observed samples and compares it to a threshold *γ*:

2 $$\begin{aligned} T(y_s)\;\underset{\mathcal {H}_0}{\overset{\mathcal {H}_1}{\gtrless }}\;\gamma . \end{aligned}$$

Methods differ primarily in the choice of *T(· )*; for example, energy detection uses received energy, cyclostationary sensing exploits spectral correlation, and eigenvalue-based methods use covariance-matrix structure.

For energy detection, sampling at rate $f_s$ over an observation interval $[0,T_0]$ produces $N=T_0 f_s$ samples $y[n]=y_s(nT_s)$, yielding the discrete-time statistic

3 $$\begin{aligned} T_{\mathrm{ED}}\approx \frac{1}{N}\sum _{n=1}^{N}|y[n]|^2 , \end{aligned}$$

which we adopt in our simulations.

Under these assumptions, with *j*(*t*) absorbed into an elevated but deterministic effective noise floor $\sigma _{\mathrm{eff}}^2 = \sigma ^2 + \sigma _j^2$ under $\mathcal {H}_0$, the scaled ED statistic follows a chi-square law under $\mathcal {H}_0$ and a noncentral chi-square law under $\mathcal {H}_1$^35,36^, as is standard in energy detection analysis. Consequently, the false-alarm and detection probabilities can be expressed in terms of the corresponding CDFs,

4 $$\begin{aligned} \begin{aligned} P_{\mathrm{FA}}&= \Pr \!\left\{ T_{\mathrm{ED}} \ge \gamma \mid \mathcal {H}_0 \right\} , \\ P_{\mathrm{D}}&= \Pr \!\left\{ T_{\mathrm{ED}} \ge \gamma \mid \mathcal {H}_1 \right\} , \end{aligned} \end{aligned}$$

and are often approximated using Gaussian (CLT) expressions for large *N* when closed-form threshold selection is desired.

A fundamental weakness of conventional ED is its reliance on accurate knowledge of the noise power: even small uncertainty can prevent reliable detection below a threshold SNR, leading to the well-known “SNR wall” phenomenon^35^. Matched-filter (MF) detection is optimal in AWGN when the PU waveform is perfectly known^36^; however, this requirement (modulation, synchronization, pulse shaping, etc.) is rarely satisfied in opportunistic access settings, so MF is typically regarded as a theoretical performance upper bound rather than a deployable sensing method^2^.

Cyclostationary feature detection exploits periodic statistics in modulated signals (absent in stationary noise) through spectral correlation measurements^37^. It is notably robust to NU and can operate at lower SNRs than energy detection; however, this robustness comes at the cost of longer observation intervals and higher computational complexity.

Eigenvalue-based detection is another blind approach that leverages the structure of the sample covariance matrix $\textbf{R}=\frac{1}{N}\textbf{y}\textbf{y}^H$^38^. A common test statistic is the ratio of the largest eigenvalue to the average eigenvalue, for example:

5 $$\begin{aligned} T_{\mathrm{EVD}} = \frac{\lambda _{\max }}{\frac{1}{K}\sum _{i=1}^{K}\lambda _i}, \end{aligned}$$

which is less sensitive to the absolute noise level because it relies on eigenvalue relationships rather than raw energy. The drawback of eigenvalue-based sensing is its high computational cost—covariance estimation and eigen-decomposition become burdensome for large signal dimensions *K* or wideband scenarios^39^.

### Noise uncertainty and jamming attacks

Noise uncertainty (NU) refers to a mismatch between the true background noise power and the value assumed during threshold setting^35^. In fixed-threshold energy detection, even a small mismatch can cause the actual false-alarm rate to diverge from the intended value, potentially creating an SNR wall below which reliable detection is impossible^35,40^. Adaptive thresholding mitigates this issue by continuously estimating the local noise-plus-interference level instead of relying on a fixed noise estimate.

Beyond NU, contested spectrum operation is dominated by *structured* and often *time-varying* interference (including intentional jamming) that corrupts sensing statistics and, critically for CFAR, contaminates the reference window used to form the background estimate. Surveys provide general taxonomies of jamming behaviors and anti-jamming strategies^3^. In this paper, the specific jamming techniques evaluated (wideband barrage and swept-FM), as well as the administrator-controlled comb-sweep counter-access signal, are defined and parameterized directly in the performance analysis sections where their impacts are measured (see “Simulation setup and performance under wideband interference”–“Performance under sweep-FM jamming” and “Administrator-controlled counter-access via comb-sweep jamming” sections).

While interference is conventionally treated as purely detrimental in spectrum sensing, recent work in machine learning has shown that certain structured perturbations can paradoxically enhance inference by reducing task complexity and suppressing irrelevant information^41,42^. This perspective offers a complementary interpretation of hybrid CFAR mechanisms: methods such as FOD-CFAR and OS-based censoring do not simply discard interference—they implicitly exploit the statistical structure of contamination to isolate the clean reference subset and improve threshold estimation. The role of noise is therefore context-dependent, a theme that also underlies the security paradox examined in “The security paradox: when robustness becomes vulnerability” section.

## System model and frequency-domain CFAR framework

CFAR detection originated in radar, where a detection statistic in each range—Doppler cell is compared to an adaptive threshold maintaining constant false-alarm rate. Recent work^14^ adapted this to cognitive radio frequency-domain sensing: the SU partitions the spectrum into resolution cells and computes a test statistic $T(f_k)$ for each cell using an adaptive threshold for PU detection. This framework preserves the constant-$P_{\mathrm{fa}}$ principle while remaining blind and scalable to wideband sensing. That analysis evaluated five classical CFAR variants under barrage and swept-FM jamming, identifying SO-CFAR and Censored-CFAR as most resilient.

### Fixed-threshold vulnerability and the frequency-domain CFAR framework

Fixed-threshold energy detection is simple but fragile: once an interference component *j*(*t*) elevates the effective background power, the actual false-alarm probability grows far beyond the design target *α*,

6 $$\begin{aligned} \mathbb {P}\!\left[ T>\gamma \,\big |\, \mathcal {H}_0+j(t) \right] \gg \alpha , \end{aligned}$$

effectively creating denial-of-service for the SU even when the PU link remains fully operational. This false-alarm inflation motivates adaptive thresholding, as demonstrated in^14^ for an APCO P25 system under broadband interference at ISR *= -20* dB.

CFAR detection addresses this by estimating the local noise-plus-interference floor directly from the received signal and scaling the threshold accordingly. In the frequency-domain framework of^14^, the SU computes an *N*-point FFT periodogram and partitions the spectrum into non-overlapping cells, each aggregating $M = \lceil B_{\mathrm{PU}}/B_{\mathrm{FFT}} \rceil$ consecutive bins matched to the PU bandwidth. The average power in cell *c*,

7 $$\begin{aligned} \bar{P}_c = \frac{1}{M} \sum _{k=k_c}^{k_c+M-1} P[k], \end{aligned}$$

serves as the test statistic. A sliding window centered on the cell under test (CUT) uses surrounding reference cells—separated from the CUT by guard cells to prevent signal leakage—to form a noise estimate $Z_{c_0}$. The adaptive threshold $\lambda _{c_0} = \alpha Z_{c_0}$ then maintains a constant false-alarm rate regardless of the interference level, with the decision rule $\bar{P}_{c_0} \gtrless \lambda _{c_0}$. All CFAR variants operate on the same frequency-domain sensing framework established in^14^: the SU computes an FFT-based periodogram, partitions it into cells matched to PU bandwidth, and slides a CFAR window over the cell-power sequence.

### Hybrid CFAR architectures in the frequency domain

Classical CFAR variants (CA, GO, SO, OS, Censored) use fixed rules to compute $Z_{c_0}$: CA-CFAR averages all reference cells, SO-CFAR takes the minimum of left/right averages, OS-CFAR selects a fixed rank, and Censored-CFAR removes a predetermined number of extreme values. These methods work well when interference is either absent or uniformly distributed across the reference window. However, when interference exhibits *spatial structure*—meaning contamination affects only part of the reference window (e.g., one side, scattered outliers, or specific subregions)—fixed rules can fail: averaging includes corrupted samples (inflating $Z_{c_0}$), while aggressive censoring may discard clean samples. Hybrid CFAR architectures address this by *adapting* the estimator $Z_{c_0}$ based on observed reference-cell characteristics. Rather than applying one fixed rule, hybrids first analyze the reference window to infer the spatial pattern of contamination, then select or combine estimation strategies accordingly. This adaptation enables robust performance across heterogeneous interference conditions that would cause classical variants to fail.

It should be noted that all eleven hybrid CFAR variants evaluated in this paper are adopted from existing radar literature^13,20–28^ and are not proposed by the authors. The authors’ contribution lies in: (i) grouping these variants into four coherent families by adaptation mechanism; (ii) adapting them from time-domain radar processing to the frequency-domain cognitive radio sensing framework established in^14^; (iii) re-calibrating their scaling thresholds $T_\alpha$ via simulation to maintain $P_{\mathrm{fa}} = 0.1$ under the AWGN/jamming conditions of this paper; and (iv) providing the first systematic comparative evaluation of these hybrid architectures against classical baselines under cognitive radio jamming conditions. Individual variant citations are provided in the algorithm pseudocode blocks (Algorithms 1–4) for precise attribution.

We evaluate eleven hybrid architectures, which we group into four families by their adaptation mechanisms. All use $N_g=1$ guard cell and $N_r=8$ reference cells per side. We denote left references as $\{L_i\}_{i=1}^{N_r}$ and right references as $\{R_i\}_{i=1}^{N_r}$.

#### Family I: adaptive algorithm selection

These hybrids treat the reference window as a classification problem. They compute *side statistics*—homogeneity metrics calculated separately for left and right reference halves (mean $\mu _L, \mu _R$; variance $\sigma _L^2, \sigma _R^2$; variability indices)—then *switch* among classical estimators (CA, GO, SO) based on detected patterns. For example, if both sides appear homogeneous, use CA; if one side shows high variability (suggesting contamination), use SO to select the cleaner side; if the mean ratio suggests an edge, use GO. This family is effective when interference is *localized* or *one-sided*, as detailed in Algorithm 1.

**Algorithm 1:**
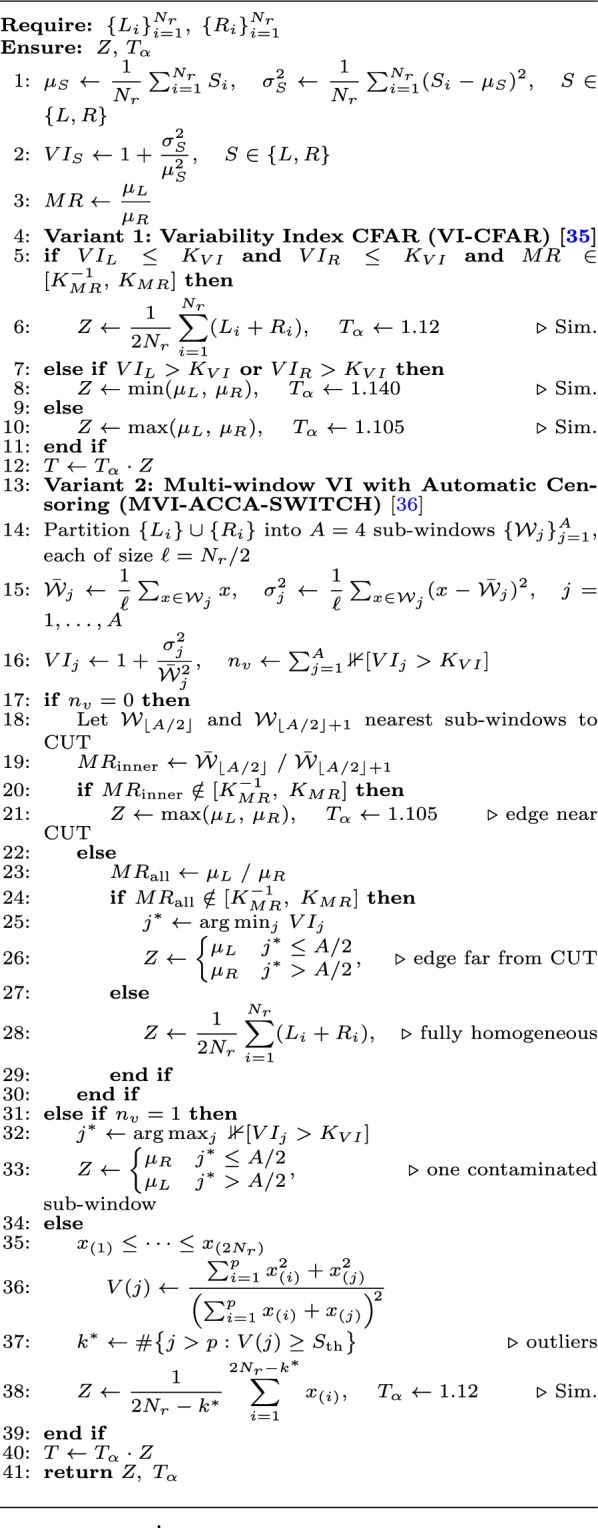
Adaptive algorithm selection (VI-CFAR, MVI-ACCA-SWITCH).

Variability Index CFAR (VI-CFAR) uses fixed thresholds $K_{VI} \approx 0.3$, $K_1 \approx 0.5$, $K_2 \approx 2.0$ tuned for $P_{fa}=0.1$. The algorithm walks through three decision branches: first checking if both sides are homogeneous (low variability on both), then checking if either side shows contamination (high variability), and finally detecting edge conditions via mean ratio. Multi-window VI with Automatic Censoring (MVI-ACCA-SWITCH) increases granularity by partitioning each side into two sub-windows. When contamination is scattered across multiple sub-windows ($n_v \ge 2$), it invokes iterative Automatic Censoring-Ordered Data Variability (ACCA-ODV) censoring that progressively removes outliers. The ODV statistic $V_k$ computes a normalized energy ratio of the first *k* sorted samples: a large $V_k$ indicates that the *k*-th sample is disproportionately large relative to the running set, flagging it as an outlier and triggering its removal. This process repeats until $V_k$ stabilizes, indicating all remaining samples are from the noise-only distribution.

#### Family II: order-statistic fusion

Instead of averaging (which is sensitive to outliers), these hybrids extract a *rank-based* representative from each side. They sort left and right references independently, select the *k*-th order statistic from each (typically $k = \lfloor 3N_r/4 \rfloor = 6$ for $N_r=8$, which suppresses up to 25% outliers per side), then fuse the two side-estimates using max Order Statistic Greatest-Of (OSGO) or min Order Statistic Smallest-Of (OSSO). Because the estimate is driven by rank rather than mean, these methods suppress a limited number of strong outliers and are especially useful when only one side of the reference window is contaminated, as shown in Algorithm 2.

**Algorithm 2:**
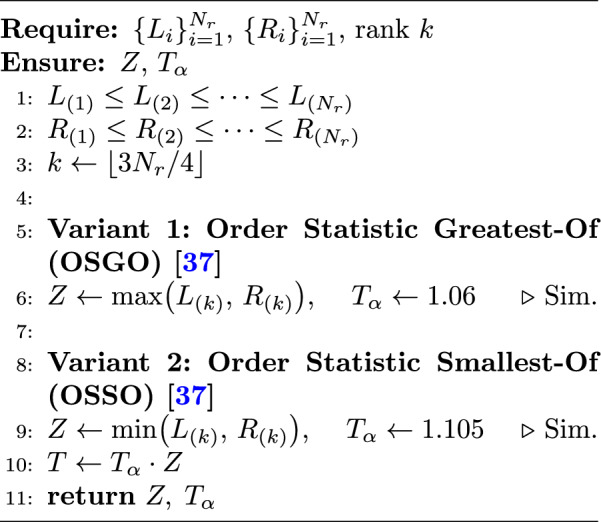
Order-statistic fusion (OSGO-CFAR, OSSO-CFAR).

The algorithm begins by sorting each side’s references to establish the empirical cumulative distribution. The key insight is that the *k*-th order statistic ($k < N_r$) provides a robust central tendency measure that automatically discards the highest values. For *k = 6* out of $N_r=8$, the estimate is the 6th smallest (3rd largest) value, meaning up to 2 outliers per side (25%) are automatically suppressed. The rank *k* trades robustness versus sensitivity: higher *k* (e.g., $k=N_r-1$) is more conservative, resisting outliers but potentially overestimating noise; lower *k* (e.g., $k=N_r/2$) is more sensitive but vulnerable to strong outliers. After extracting order statistics from both sides, OSGO selects the maximum (more conservative, suitable when both sides may be contaminated), while OSSO selects the minimum (optimistic, assumes at least one side is clean). The fusion step effectively implements a “best-case” (OSSO) or “worst-case” (OSGO) estimate based on which side is cleaner.

#### **Family III: intelligent censoring and jump rejection**

These hybrids explicitly identify and *remove* outliers before averaging. Unlike fixed censoring (which removes a predetermined number of cells), intelligent censoring uses statistical tests to decide which cells are contaminated. Methods include: First-Order Difference (FOD), which detects statistical jumps in sorted sequences; Auto-Censored Greatest-Of CA (ACGCA), which applies iterative censoring with a stopping criterion; Auto Dual-threshold Censored CA (ADCCA), which uses fuzzy membership functions; Median-Scaled Spike Suppression (MSS), which performs median-based spike detection; and Clutter Map—Censored Mean (CM-CM), which combines temporal and spatial censoring. This family is most beneficial when interference is *bursty*, *sparse*, or *structured* (e.g., comb-like), where a reject-then-average strategy better tracks the true local noise floor than raw averaging. Algorithm 3 presents five variants within this family.

**Algorithm 3:**
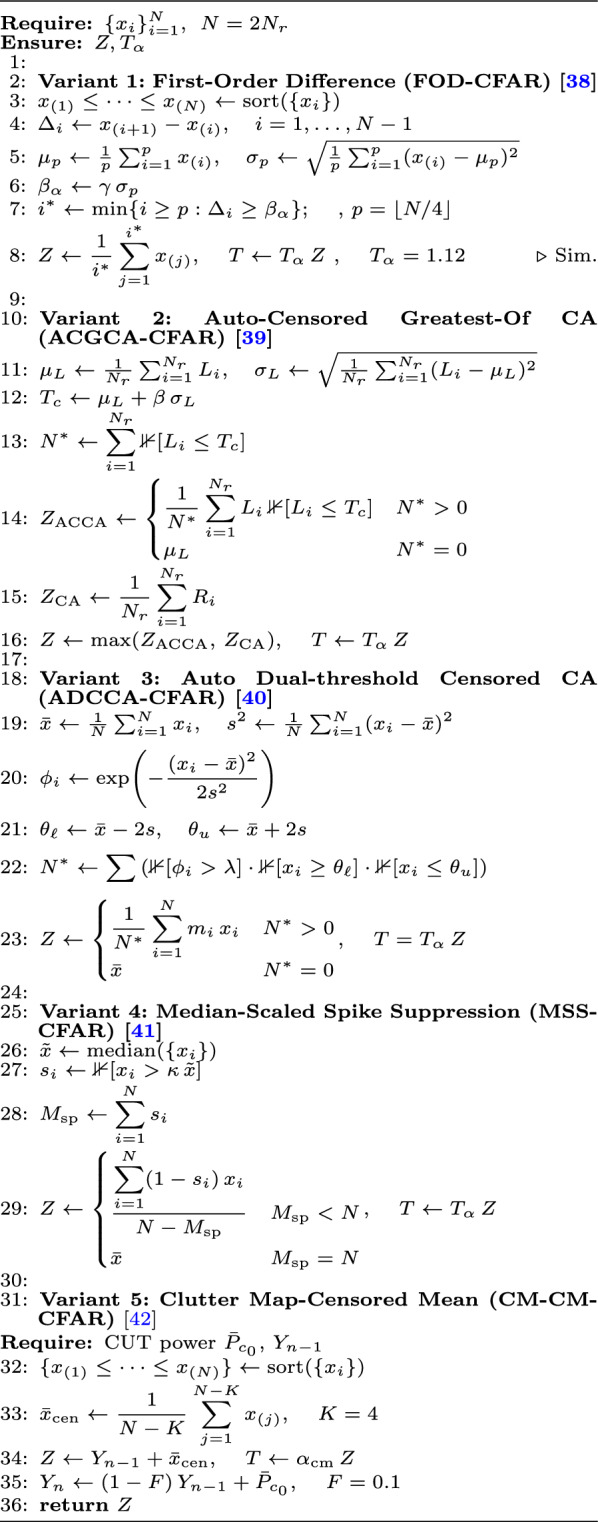
Intelligent censoring (FOD, ACGCA, ADCCA, MSS, CM-CM).

FOD-CFAR exploits the observation that when interference is present, the sorted reference sequence exhibits a discontinuity: low-power noise samples cluster at the beginning, followed by a statistical jump to high-power interference samples. The algorithm walks through the sorted sequence computing adjacent differences $\Delta _i$; when $\Delta _i$ exceeds threshold $\beta _\alpha$, the jump is detected and averaging uses only samples below this boundary. This is optimal when interference forms a distinct high-power cluster separated from noise by a clear gap. ACGCA uses the left (leading) window as a statistical reference to set an adaptive censoring threshold $T_c = \mu _L + \beta \sigma _L$; values exceeding this threshold are rejected from both sides before computing side means. The subsequent Greatest-Of fusion provides additional robustness when one side remains contaminated after censoring. ADCCA employs fuzzy logic: instead of hard thresholds, it assigns continuous membership weights via a Gaussian kernel, giving high weight to samples near the mean and low weight to outliers. Extreme outliers beyond $[\mu - 2\sigma , \mu + 2\sigma ]$ are still hard-rejected. This smooth weighting avoids abrupt censoring decisions and is robust to borderline outliers. MSS takes a different approach by using the median $\tilde{x}$ (inherently robust to outliers) as a reference point, then flagging samples that exceed $\kappa \cdot \tilde{x}$ (typically *κ =3*) as outliers and excluding them from the noise estimate. This is simple and effective against isolated strong interferers. CM-CM combines two censoring stages: temporal filtering via exponential smoothing tracks slow variations across frequency sweeps, while spatial censoring removes the top 25% of current references. The combination of memory and spatial robustness makes this variant suitable for slowly-varying interference patterns.

#### **Family IV: weighted and partitioned processing**

These hybrids reshape the contribution of reference cells to reduce bias from non-uniform interference across the window. Weighted Cell-Averaging (WCA-CFAR) assigns distance-based weights to emphasize near-CUT references (cells closer to the CUT are more representative of local conditions) and uses weighted moments to build the threshold. Cell-Averaging Statistic Hofele (CASH-CFAR) partitions the reference set into sub-regions (registers) and fuses extreme subwindow sums to control the impact of localized contamination. This family is useful when interference varies gradually across frequency or when localized corruption can be isolated by partition-level fusion. The two variants are detailed in Algorithm 4.

**Algorithm 4:**
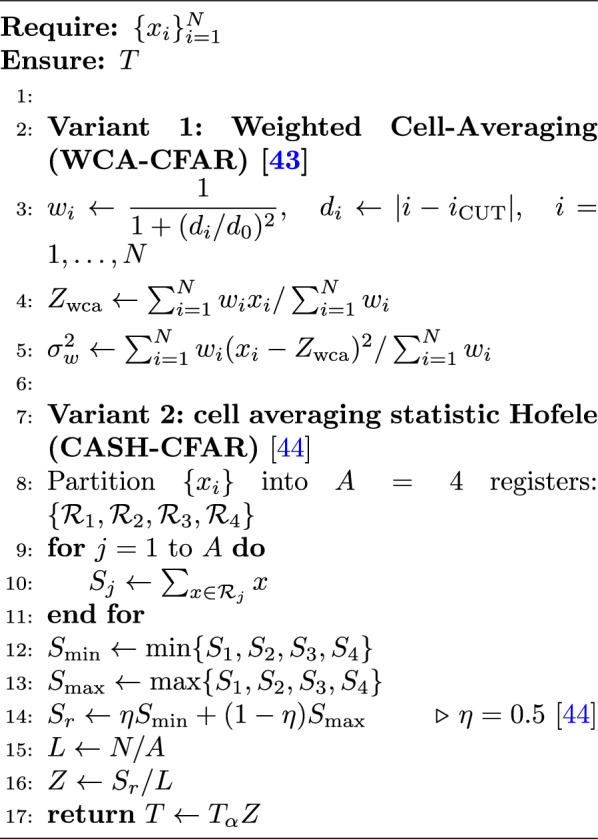
Weighted/partitioned processing (WCA-CFAR, CASH-CFAR)

WCA-CFAR begins by computing the distance $d_i$ of each reference cell from the CUT position. The weighting function $w_i = 1/(1+(d_i/d_0)^2)$ implements an inverse-square decay: cells immediately adjacent to guard cells receive highest weight (near 1.0), while distant cells are downweighted. The distance scale parameter $d_0$ controls the weighting profile—smaller $d_0$ gives sharper decay (emphasizing immediate neighbors), larger $d_0$ gives flatter weighting (approaching uniform CA). After computing weights, the algorithm forms a weighted mean $Z_{\text {wca}}$ which emphasizes local conditions near the CUT. The weighted variance $\sigma _w^2$ similarly emphasizes local spread, and the threshold is adjusted by adding $1.6\sqrt{\sigma _w^2}$ to account for local noise variability. This dual-moment approach (mean plus spread adjustment) provides adaptive thresholding that tracks both level and dispersion. cell averaging statistic Hofele (CASH-CFAR) takes a partitioning approach: it divides the reference window into *A=4* equal-sized registers and computes the sum within each partition. The algorithm then identifies the minimum sum $S_{\min }$ (representing the cleanest partition, least affected by interference) and maximum sum $S_{\max }$ (representing the most contaminated partition). Rather than using either extreme alone, CASH fuses them via weighted combination $S_r = \eta S_{\min } + (1-\eta )S_{\max }$. The fusion parameter *η* trades between conservative ($\eta \rightarrow 0$, favoring the contaminated partition’s sum) and optimistic ($\eta \rightarrow 1$, favoring the clean partition’s sum); typical choice is *η = 0.5* for balanced performance. The fused sum is then normalized by register size to obtain the noise estimate. This partitioning strategy isolates localized contamination to specific registers, preventing it from biasing the entire estimate.

## Resilience of hybrid frequency-domain CFAR under heterogeneous interference and security-oriented threats

This section evaluates the hybrid CFAR architectures from “Hybrid CFAR architectures in the frequency domain” section. Our previous work^14^ established baseline performance of classical variants under jamming. We examine how hybrid estimators improve robustness against *structured* and *time-varying* interference by adapting $Z_{c_0}$ based on reference-window characteristics.

Performance is quantified using (i) probability of detection $P_{\mathrm{d}}$ versus SNR and (ii) false-alarm behavior $P_{\mathrm{fa}}$ summarized via distributional statistics (median and interquartile range) across Monte Carlo trials. We consider two operational threat models in this section: (a) barrage (wideband) jamming as a sanity-check baseline and (b) swept-FM jamming with increasing spectral coverage as a controlled stress test that exposes algorithm-specific breaking points. The final subsection revisits the *security paradox*: increased CFAR robustness improves SU survivability under hostile conditions, but simultaneously weakens spectrum governance by resisting conventional denial signals, motivating the administrator-controlled comb-sweep counter-access strategy analyzed in “Administrator-controlled counter-access via comb-sweep jamming” section.

### Simulation setup and performance under wideband interference

Monte Carlo simulations were conducted using APCO Project 25 (P25) PUs in a 50-channel spectrum observed by an OFDMA-based SU, with an AWGN channel and NU of 0 dB; CFAR’s inherent robustness to NU follows directly from its adaptive estimation of $Z_{c_0}$, as established in “Noise uncertainty and jamming attacks” section. Jammer strength is measured by JSR, and for swept jammers total jammer power is held constant across tests (power per channel decreasing proportionally as coverage increases), isolating reference-window contamination effects from raw jammer power. All detectors share the decision rule $\bar{P}_{c_0}\gtrless \lambda _{c_0}$ with $\lambda _{c_0}=\alpha Z_{c_0}$, differing only in how $Z_{c_0}$ is formed; hybrid-specific parameters (ranks, censoring levels, switching thresholds, weighting coefficients) are held fixed at their standard definitions from “Hybrid CFAR architectures in the frequency domain” section across all scenarios, so that performance changes are attributable to the interference environment rather than retuning. Performance is evaluated across three jamming regimes of increasing structure: wideband barrage jamming (this subsection), swept-FM jamming with tunable spectral coverage (“Performance under sweep-FM jamming” section), and administrator-controlled comb-sweep jamming for security analysis (“Administrator-controlled counter-access via comb-sweep jamming” section).

For comparison, we include an exponential smoothing noise tracker (ES-Tracker) as a non-CFAR adaptive baseline. The ES-Tracker applies a first-order IIR filter directly to the CUT energy to estimate the noise floor,

8 $$\begin{aligned} \hat{Z}_n = (1-\alpha )\,\hat{Z}_{n-1} + \alpha \,\bar{P}_{c_0}, \quad \lambda = T_\alpha \,\hat{Z}_n, \end{aligned}$$

with smoothing factor *α = 0.1* and threshold scaling $T_\alpha = 1.12$ matched to the target $P_{\mathrm{fa}} = 0.1$. Unlike CFAR, which estimates the noise floor exclusively from spatial reference neighbors by explicitly excluding the CUT via guard cells, the ES-Tracker updates its estimate from the CUT itself. In the P25 trunked waveform environment used in this paper, where channels appear and disappear rapidly and frequently, this causes the tracker to confound signal energy with noise, producing a threshold that follows channel activity rather than the true noise floor.

**Fig. 1:**
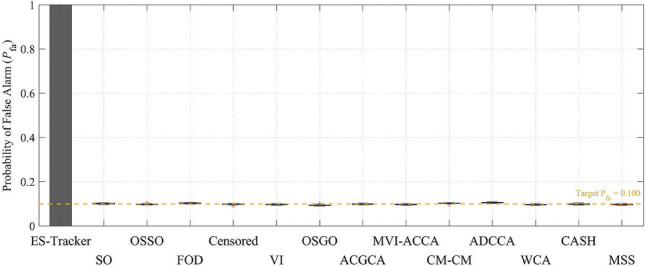
$P_{\mathrm{fa}}$ under barrage jamming ($\mathrm{JSR} = -10$ dB): all thirteen hybrid CFAR variants concentrate at the design target $P_{\mathrm{fa}} = 0.1$, while the ES-Tracker spans nearly the full range [0, 1].

We use $\mathrm{JSR}=-10$ dB as a baseline to verify false-alarm control and detection convergence for all hybrid CFAR architectures, alongside the SO-CFAR and Censored-CFAR baselines from^14^, which demonstrated superior performance among classical variants. As shown in Fig. 1, all hybrid CFAR variants maintain the target false-alarm rate of 0.1 under homogeneous wideband interference, confirming that the CFAR property is preserved under elevated interference—a necessary precondition for the detection-probability results that follow^9^. In contrast, the ES-Tracker’s $P_{\mathrm{fa}}$ spans nearly the full range [0, 1] across Monte Carlo trials, confirming that temporal averaging of the CUT energy cannot maintain a controlled false-alarm rate when signal activity is non-stationary.

**Fig. 2:**
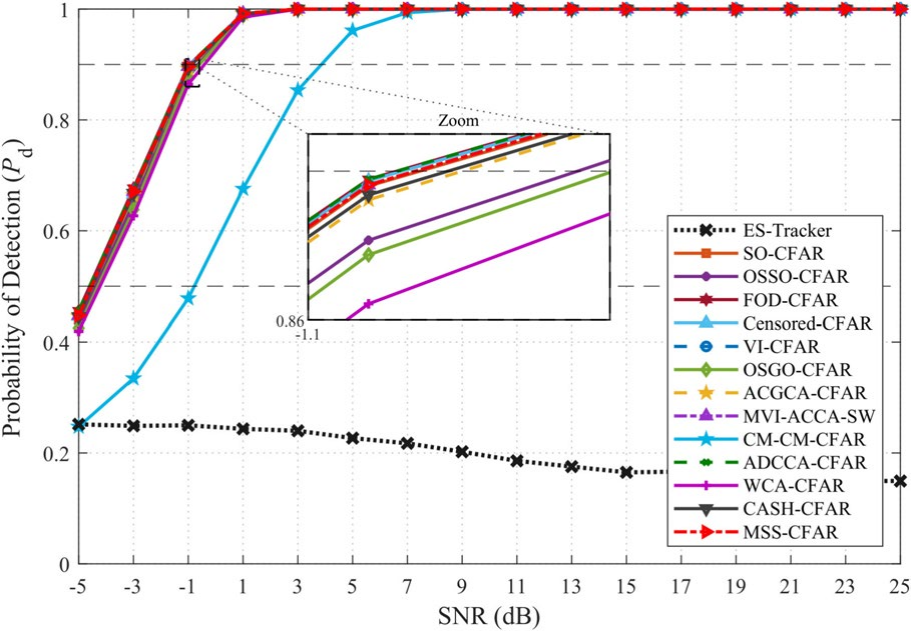
$P_{\mathrm{d}}$ vs. SNR under barrage jamming ($\mathrm{JSR} = -10$ dB): the ES-Tracker remains entirely below the operational detection region ($P_{\mathrm{d}} < 0.5$) across the full SNR range, while all thirteen hybrid CFAR variants reach $P_{\mathrm{d}} \approx 1$ by SNR *= 1* dB.

Figure 2 presents detection performance under the same barrage conditions. The shaded region between $P_{\mathrm{d}}=0.5$ and $P_{\mathrm{d}}=0.9$ indicates the operational transition region. Nearly all hybrid CFARs traverse this region rapidly, reaching $P_{\mathrm{d}} \approx 1$ by SNR *= 0*–1 dB. The exception is CM–CM-CFAR, which requires approximately 7 dB to achieve near-unity $P_{\mathrm{d}}$ due to its conservative memory-assisted estimation under uniformly elevated interference. No hybrid fails catastrophically; all methods eventually saturate at high $P_{\mathrm{d}}$, confirming robustness when interference is unstructured and evenly distributed. The ES-Tracker, by contrast, remains below the operational region across the full SNR range, a direct consequence of its uncontrolled threshold: because the tracker absorbs signal energy into its noise estimate when the P25 channel is active, the threshold is set inconsistently across trials, preventing reliable detection convergence. In summary, barrage jamming serves as a non-discriminative baseline for the CFAR suite: all hybrids preserve $P_{\mathrm{fa}}=0.1$ and reach $P_{\mathrm{d}}\approx 1$ by SNR *=1* dB (except CM–CM-CFAR which saturates near SNR *=7* dB), while the ES-Tracker confirms that spatial reference-window estimation is architecturally essential for maintaining detection reliability in this environment.

### Performance under sweep-FM jamming

Before examining hybrid performance, we briefly restate the classical baseline results from^14^ for direct comparison. Under swept-FM jamming, SO-CFAR achieved $P_{\mathrm{d}} \approx 1$ by SNR *= 1* dB at light coverage (10%) and degraded to $P_{\mathrm{d}} \approx 0.8$ at moderate coverage (40%), remaining at the lower edge of the operational detection region. Censored-CFAR followed similarly at light coverage ($P_{\mathrm{d}} \approx 0.9$) but fell outside the operational detection region at moderate coverage ($P_{\mathrm{d}} \approx 0.4$). These figures represent the performance ceiling of classical variants and serve as the benchmark against which the hybrid architectures are evaluated across the four coverage regimes discussed below.

A contiguous jammer footprint of $M_J$ channels traverses the *C=50* sensing cells, creating alternating clean and contaminated reference-window realizations.

As the sweep jamming becomes more concentrated (covering fewer than all 50 channels at once), the effective per-channel JSR increases. For example, jamming $M_J=25$ channels instead of 50 raises the JSR per channel from *-10* dB to approximately 0 dB. This occurs because concentrating the same total jammer power over fewer channels increases the power density per channel, following

9 $$\begin{aligned} \mathrm{JSR}_{\mathrm{eff}}(M_J) = \mathrm{JSR}_{\mathrm{barr}} + 10\log _{10}\!\left( \frac{C}{M_J/5}\right) , \end{aligned}$$

where $M_J$ is the sweep coverage in channels, *C = 50* is the total band, and the factor 5 arises from the ratio of the SU sensing interval to the effective jammer dwell time per channel:

10 $$\begin{aligned} \begin{gathered} \frac{T_0}{T_{\mathrm{dwell}}} = \frac{N/f_s}{1/B_{\mathrm{ch}}} = \frac{1024\,/\,2.4576\times 10^6}{1\,/\,12500} \\ \approx \frac{416~\mu \text {s}}{80~\mu \text {s}} \approx 5. \end{gathered} \end{aligned}$$

**Fig. 3:**
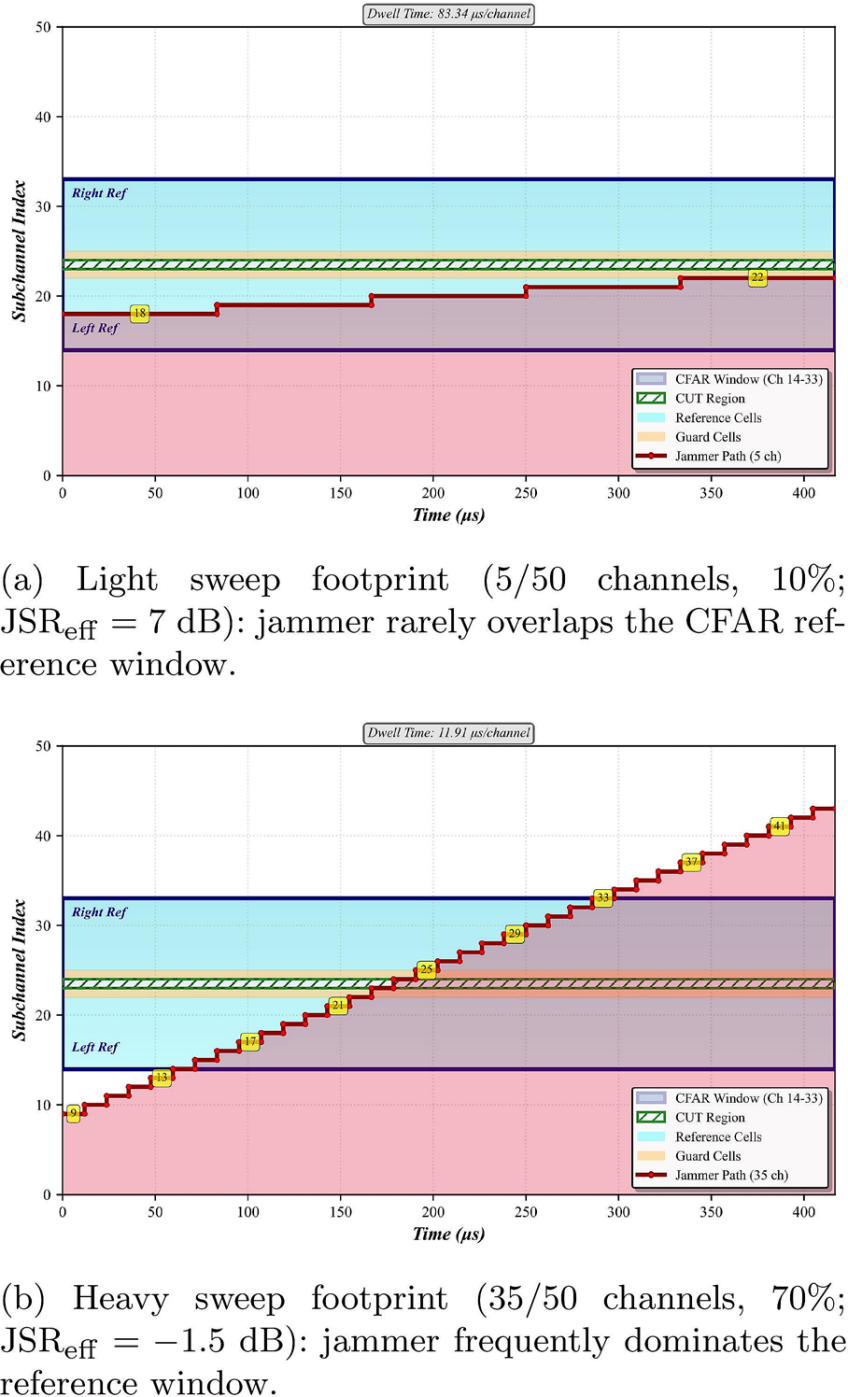
Time–frequency illustration of FM sweep jamming at the two extreme coverage levels (light vs. heavy), contrasting nearly clean against severely contaminated reference-window realizations. Higher coverage raises contamination probability while reducing effective per-channel JSR through faster sweeping.

This dwell-time ratio captures the sweep rate’s impact on per-channel interference density. Since both $T_0$ and $T_{\mathrm{dwell}}$ are determined solely by the fixed P25 waveform parameters (*N = 1024*, $f_s = 2.4576$ MHz, $B_{\mathrm{ch}} = 12.5$ kHz), the factor of 5 remains constant throughout all sweep-FM experiments in this paper. Figure 3 illustrates the two extreme sweep footprints to make the contrast between clean and heavily contaminated reference-window realizations visually apparent. We evaluate four coverage levels: light (10%, $M_J=5$, $\mathrm{JSR}_{\mathrm{eff}}=7$ dB), moderate (40%, $M_J=20$, $\mathrm{JSR}_{\mathrm{eff}}=1$ dB), critical (50%, $M_J=25$, $\mathrm{JSR}_{\mathrm{eff}}=0$ dB), and heavy (70%, $M_J=35$, $\mathrm{JSR}_{\mathrm{eff}}=-1.5$ dB). Increasing coverage simultaneously (i) increases the probability of reference contamination and (ii) reduces the effective per-channel JSR due to faster sweeping.

We now discuss sweep jamming performance across four representative coverage regimes.

**Fig. 4:**
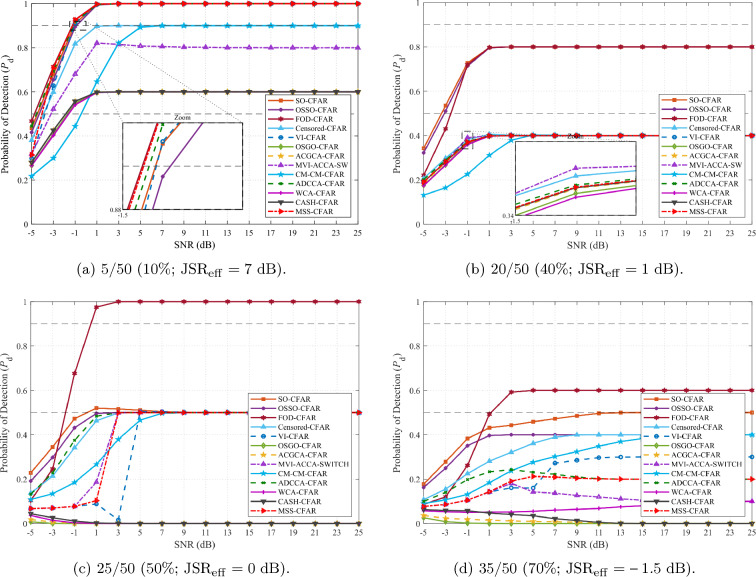
$P_{\mathrm{d}}$ vs. SNR under FM sweep jamming across all four coverage levels (10–70%).

Figure 4a shows that at light coverage (10%), jammer interference rarely overlaps the CFAR reference window, yet performance stratification already emerges across four distinct groups. The first group—comprising SO, OSSO, FOD, VI, ADCCA, and MSS—saturates at $P_{\mathrm{d}} \approx 1$ by SNR *= 1* dB, maintaining performance comparable to the barrage baseline. The second group, Censored-CFAR and CM-CM-CFAR, saturates at $P_{\mathrm{d}} \approx 0.9$ by SNR *= 1* dB and 5 dB, respectively, reflecting their inherent conservatism under transient contamination. The third group, MVI-ACCA-SWITCH, saturates at $P_{\mathrm{d}} \approx 0.8$ at SNR *= 1* dB due to its multi-window partitioning triggering premature censoring decisions. The fourth group—OSGO, ACGCA, WCA, and CASH—saturates at $P_{\mathrm{d}} \approx 0.6$ at SNR *= 1* dB, indicating these methods are overly conservative even under sparse contamination, elevating thresholds beyond necessary levels.

Figure 4b shows that at moderate coverage (40%), contamination becomes frequent enough that performance differences sharpen into two clear groups. FOD, SO, and OSSO saturate at $P_{\mathrm{d}} \approx 0.8$ by SNR *= 1* dB, remaining within the operational region. FOD’s advantage stems from its global sorting and jump detection, which isolates clean reference cells regardless of contamination distribution. SO and OSSO benefit from their side-selection mechanisms (choosing the minimum of left/right estimates), which effectively discard the contaminated side when interference is asymmetric. In contrast, all other variants saturate at $P_{\mathrm{d}} \approx 0.4$, falling outside the operational region. These methods either average contaminated references (inflating $Z_{c_0}$) or apply overly conservative fusion rules that elevate thresholds excessively. This regime marks the onset of significant hybrid differentiation.

Figure 4c demonstrates that at critical coverage (50%), it is common for one entire half of the reference window to be heavily contaminated while the other remains comparatively clean, representing a fundamental breaking point for side-based estimators. FOD-CFAR consistently outperforms estimators that process left and right reference halves separately (e.g., SO, OSSO, GO, OSGO). Mechanistically, FOD operates on a *globally sorted* reference set and detects the first significant statistical jump separating noise-dominated cells from jammer-dominated cells. As long as a nontrivial clean subset exists, FOD forms $Z_{c_0}$ from the clean cells regardless of whether contamination is concentrated on the left or right side. In contrast, side-based estimators effectively lose usable support when both sides become corrupted. At SNR *= 3* dB, FOD achieves $P_{\mathrm{d}} \approx 1$, compared to $P_{\mathrm{d}} \approx 0.5$ for SO and OSSO. Importantly, all methods maintain false-alarm control under $\mathcal {H}_0$ despite the asymmetric contamination.

Figure 4d shows that under heavy coverage (70%), clean reference cells become scarce for many sweep positions, and all methods degrade because $Z_{c_0}$ is increasingly biased by jammer-contaminated references. FOD remains best-in-class but with diminishing advantage as the clean-cell subset frequently falls below the minimum needed for stable estimation. At SNR *= 3* dB, FOD saturates at $P_{\mathrm{d}} \approx 0.60$ and SO-CFAR sits at the lower edge of the operational region, while all other variants fall outside it. This regime highlights a fundamental limit: once contamination dominates the reference window most of the time, even robust hybrid mechanisms cannot guarantee sufficient clean samples to sustain high detection.

Taken together, the four coverage regimes reveal a clear and consistent performance hierarchy that sharpens progressively with jamming intensity. At light coverage, most hybrids perform comparably, with only the most conservative variants (OSGO, ACGCA, WCA, CASH) showing early degradation. As coverage increases to moderate and critical levels, the field narrows: FOD-CFAR separates itself as the most resilient, followed by SO-CFAR and OSSO-CFAR, while the remaining variants fall outside the operational detection region. Under heavy coverage, even the leading group degrades, though FOD retains a measurable advantage by virtue of its global ordering strategy. This progression underscores that no single hybrid is uniformly optimal across all conditions—algorithm choice must account for the expected jammer footprint—and that global, jump-based estimation (FOD) offers the broadest robustness as the interference structure becomes more severe.

A note on the practical limits of FOD-CFAR is warranted. When no first-order difference $\Delta _i$ exceeds the jump threshold $\beta _\alpha$—as under barrage jamming where interference is uniformly distributed across all reference cells—FOD sets $i^* = N$ and reverts to averaging all reference cells, behaving identically to CA-CFAR. This is a graceful degradation: uniform contamination is correctly absorbed into the noise estimate, as confirmed by the barrage results in “Simulation setup and performance under wideband interference” section. The failure boundary of FOD-CFAR is more precisely defined by its seed initialization: the algorithm assumes the first $p = \lfloor N/4 \rfloor$ sorted samples represent a clean noise floor. If fewer than 25% of reference cells are free of interference, the seed itself is corrupted, causing the jump threshold $\beta _\alpha = \gamma \sigma _p$ to be set unreliably and the subsequent averaging to include contaminated cells.

**Fig. 5:**
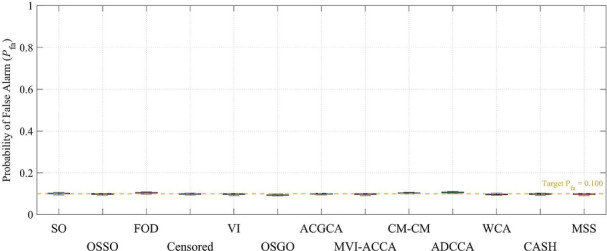
$P_{\mathrm{fa}}$ across all thirteen hybrid CFAR algorithms (x-axis) under FM sweep jamming (35/50 channels, $\mathrm{JSR}_{\mathrm{eff}}=-1.5$ dB); $P_{\mathrm{fa}} \approx 0.1$ for all sweep coverages, confirming that FM sweep degrades $P_{\mathrm{d}}$ under $\mathcal {H}_1$ while the CFAR constraint under $\mathcal {H}_0$ remains satisfied across all variants.

Unlike detection probability, which degrades with increasing sweep coverage, the false-alarm probability remains effectively invariant and tightly concentrated around the design target $P_{\mathrm{fa}} = 0.1$ for all hybrid CFARs. Figure 5 provides a representative $P_{\mathrm{fa}}$ distribution (35-channel sweep, $\mathrm{JSR}_{\mathrm{eff}} = -1.5$ dB); the remaining sweep settings are omitted because they are visually indistinguishable. The x-axis lists all thirteen algorithm names ordered for comparison, and the concentration of every bar at $P_{\mathrm{fa}} \approx 0.1$ is a significant and deliberate outcome: it confirms that FM sweep jamming primarily corrupts the reference set under $\mathcal {H}_1$ (reducing $P_{\mathrm{d}}$, as shown in Fig. 4), while the adaptive thresholding mechanism successfully preserves the CFAR constraint under $\mathcal {H}_0$ across all hybrid variants and all tested sweep coverage levels.

### Computational complexity and implementation considerations

Hybrid CFAR architectures improve detection robustness by adapting how reference cells are processed to estimate the local noise-plus-interference level $Z_{c_0}$. This adaptation adds computational overhead compared to simple averaging methods (CA/GO/SO). Since the CFAR window slides across *C* cells in each sensing interval, total cost scales as

11 $$\begin{aligned} \mathcal {C}_{\text {total}} \approx C \cdot \mathcal {C}_{\text {per-CUT}}, \end{aligned}$$

where $\mathcal {C}_{\text {per-CUT}}$ depends on the number of reference cells $N = 2N_r$. With $N_r=8$ per side, we have *N=16* cells per evaluation. The key difference among algorithms is whether they use sorting (slower) or simple passes through the data (faster).

To compare the computational cost of each hybrid method, we analyze the dominant operations required per cell evaluation. Table 1 summarizes per-CUT complexity for the eleven hybrids. Algorithms using order statistics (OSGO/OSSO) or jump/median detection (FOD, MSS, CM–CM) require sorting, giving $\mathcal {O}(N\log N)$ cost^13^. Methods based on thresholds or variability checks (VI, ACGCA, WCA, CASH, ADCCA) achieve $\mathcal {O}(N)$ through linear scans^43^. MVI-ACCA-SWITCH operates at $\mathcal {O}(N)$ when fewer than two sub-windows are flagged as contaminated ($n_v < 2$), as only linear passes for sub-window statistics and switching logic are required; it escalates to $\mathcal {O}(N\log N)$ only when $n_v \ge 2$ triggers the iterative ACCA-ODV censoring, which requires sorting.

**Table 1:** Per-CUT computational complexity of hybrid CFAR estimators ($N=2N_r=16$ reference cells).

Algorithm	Complexity
VI-CFAR	$\mathcal {O}(N)$
MVI-ACCA-SWITCH	typical $\mathcal {O}(N)$worst $\mathcal {O}(N\log N)$
OSGO-CFAR	$\mathcal {O}(N\log N)$
OSSO-CFAR	$\mathcal {O}(N\log N)$
ACGCA-CFAR	$\mathcal {O}(N)$
FOD-CFAR	$\mathcal {O}(N\log N)$
ADCCA-CFAR	$\mathcal {O}(N\log N)$
MSS-CFAR	$\mathcal {O}(N\log N)$
CM–CM-CFAR	$\mathcal {O}(N\log N)$
WCA-CFAR	$\mathcal {O}(N)$
CASH-CFAR	$\mathcal {O}(N)$

Three practical points follow. *(i) Small reference sets:* With *N=16* cells, even sorting-based methods run fast on modern hardware—sorting 16 values is trivial, so complexity is not limiting in our setup (*C=50* cells). *(ii) Wideband scaling:* For ultra bands requiring hundreds of reference cells, $\mathcal {O}(N\log N)$ methods may add noticeable latency. However, typical cognitive radio windows use modest *N* (tens of cells), making linear-cost methods necessary only under tight power or processing budgets. *(iii) Performance trade-off:* Sorting-based robustness (especially FOD’s global ordering and jump detection) provides clear gains under structured jamming, but at higher computational cost. Designers must weigh this against system constraints (latency, power, bandwidth).

All evaluated variants are feasible for real-time use in this context. Optimizations like partial selection (avoiding full sorts for *k*-th statistics) or approximate median computation can reduce runtime. CM–CM also needs memory to store past values (roughly *C* floats), which matters in embedded systems with limited RAM.

Taken together, the performance and complexity results point to FOD-CFAR as the strongest overall candidate: it achieves the best detection robustness in the mid-to-high interference-coverage regime through global ordering and jump rejection, at an $\mathcal {O}(N\log N)$ cost that remains negligible for the reference-window sizes typical in cognitive radio (*N=16* in our setup). Where lower complexity is a hard constraint, VI-CFAR and ACGCA-CFAR offer a practical alternative, trading some robustness under heavy contamination for guaranteed $\mathcal {O}(N)$ operation.

### The security paradox: when robustness becomes vulnerability

The preceding results reveal a fundamental paradox in cognitive radio governance^16^: hybrid CFAR architectures designed to resist hostile jamming also resist administrative spectrum enforcement. The same adaptive mechanisms that help legitimate SUs survive interference can enable unauthorized SUs to evade denial signals^44^.

This follows directly from the CFAR constraint. Any method that estimates $Z_{c_0}$ from reference cells satisfies

12 $$\begin{aligned} \lambda _{c_0}=\alpha Z_{c_0}, \qquad \mathbb {P}\!\left( \bar{P}_{c_0}>\lambda _{c_0}\mid \mathcal {H}_0\right) =P_{\mathrm{fa}}, \end{aligned}$$

so raising interference increases both the background estimate and the threshold proportionally. This preserves the false-alarm rate and leaves detection functional. Our barrage and sweep experiments confirm this: even under heavy contamination, robust hybrids (especially those using global ordering and jump rejection) maintain detection capability. An adversary using such a sensor can thus stay aware of spectrum conditions despite interference that would disable simpler detectors.

For administrators, attempting to deny access by masking PU signals is counterproductive—it fails against adaptive CFAR while risking harmful interference to incumbents. A better strategy is to create *deceptive occupancy* on vacant channels (elevating $P_{\mathrm{fa}}$ to force unauthorized SUs to perceive the spectrum as busy) while preserving PU detection. This requires a *counter-access strategy* that exploits the statistical structure of $Z_{c_0}$ rather than simply raising the noise floor.

The next section develops a comb-sweep jamming countermeasure that targets hybrid CFAR processing to create a controlled denial plateau while maintaining PU protection.

## Administrator-controlled counter-access via comb-sweep jamming

The security paradox identified in “The Security paradox: when robustness becomes vulnerability” section shows that raising the noise floor (e.g., barrage jamming) is ineffective against CFAR-equipped SUs, since adaptive thresholding absorbs the interference without degrading detection. Instead, we propose an administrator-controlled counter-access strategy that exploits the geometry of the CFAR reference window to create a persistent illusion of occupancy on vacant channels, forcing unauthorized SUs to perceive the spectrum as busy while leaving genuine PU signals detectable. This is realized via comb-sweep jamming, whose sparse spectral structure is designed to interact directly with hybrid estimator construction.

Table 2 summarizes the three countermeasure design goals that govern all subsequent analysis in this section.

**Table 2:** Countermeasure design goals, associated metrics, and targets.

Design goal	Metric	Target
Protect PU	$P_{d}^{(\mathrm{PU})}$	High across SNR range
Deny SU	$P_{\mathrm{fa}}^{(\mathrm{vacant})}$	*→ 1* across SNR range
Balance both simultaneously	JSR operating range	Denial plateau *[-5,-1]* dB

### Signal model and deceptive occupancy framework

The proposed counter-access strategy, termed *deceptive occupancy*, targets vacant channels: rather than suppressing detection (which risks interfering with PU transmissions), the administrator maximizes the false-alarm probability on PU-absent channels ($P_{\mathrm{fa}}^{(\mathrm{vacant})}$) while maintaining high detection probability on PU-present channels ($P_{\mathrm{d}}^{(\mathrm{PU})}$). This asymmetry is fundamental: a missed detection causes harmful interference to a protected service, whereas a false alarm merely denies access to an opportunistic user.

Deceptive occupancy is realized by exploiting the reference-window structure of hybrid frequency-domain CFAR. This is feasible because the P25 channelization and timing structure of the band under enforcement is standardized and publicly specified^45^, and the administrator, by virtue of its regulatory role, possesses operational knowledge of which specific channel plan and timing offset are active in the contested band. In practice, the center frequencies of deployed P25 systems in a given region are either available through public licensing and frequency-coordination records or readily inferred by direct spectral analysis of the observed band, further supporting the feasibility of this synchronization. The administrator-controlled jammer generates two narrowband tones that sweep periodically across unallocated spectrum. Let $f_i(t) = f_c + (i-1)\Delta f + f_{\text {sweep}}(t)$ denote the instantaneous frequency of the *i*-th tone. The time-domain jammer signal is

13 $$\begin{aligned} j(t) = \sqrt{\frac{2P_J}{N_t}} \sum _{i=1}^{N_t} \cos \,\!\bigl (2\pi f_i(t)\,t + \phi _i\bigr ), \end{aligned}$$

and its instantaneous spectrum is

14 $$\begin{aligned} J(f,t) = \sum _{i=1}^{N_t} A_J\,\delta \,\!\bigl (f - f_i(t)\bigr ), \end{aligned}$$

where $P_J$ is total jamming power, $f_c$ is the starting frequency, $\Delta f = 25B_{\mathrm{ch}}$ is the tone spacing, $f_{\mathrm{sweep}}(t)$ controls the sweep, $\phi _i$ are random phases, $\delta (\cdot )$ is the Dirac delta, and $A_J$ is the tone amplitude. The two tones maintain fixed spacing while sweeping, ensuring one targets the lower reference half and the other the upper half.

The sweep period $T_{\text {period}} \approx 416~\mu$s is matched to the SU’s FFT-based sensing interval (determined by the 12.5 kHz channel bandwidth), ensuring each sensing snapshot encounters a statistically fresh interference realization. The jammer completes four such periods per cycle, giving $T_{\text {cycle}} = 4\,T_{\text {period}} \approx 1.6$ ms, and each period executes one full sweep according to

15 $$\begin{aligned} f_{\text {sweep}}(t) = f_0 + \frac{\Delta f_{\text {sweep}}}{T_{\text {period}}} \left( t \bmod T_{\text {period}}\right) . \end{aligned}$$

**Fig. 6:**
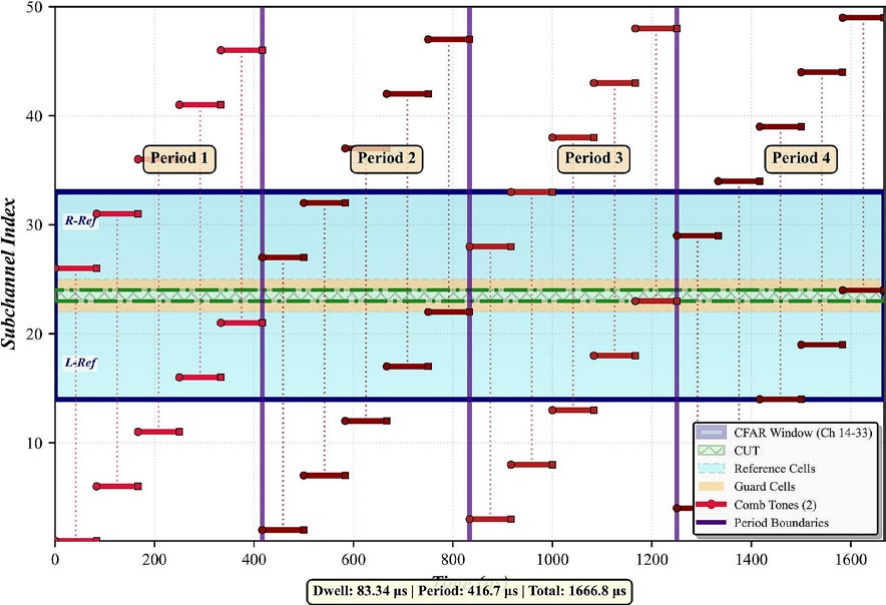
Time–frequency representation of dual-tone comb-sweep jammer over four periods (1600 *μ*s), creating structured intersections with the CFAR window.

Figure 6 illustrates the sweep pattern; parameters are in Table 3.

**Table 3:** Comb-sweep jammer configuration parameters.

Parameter	Value
Number of simultaneous tones ($N_t$)	2
Target subchannel range	1–50
($T_{\text {cycle}}$)	*≈ 1.6* ms
Number of periods	4
($T_{\text {period}}$)	416 *μ*s
CFAR window	Channels 14–33
Subchannel BW ($B_{\text {ch}}$)	12.5 kHz

When a tone occupies reference cell $j \in \mathcal {R}(c_0)$, the measured power becomes

16 $$\begin{aligned} \bar{P}_j = \bar{P}_j^{(\text {noise})} + \bar{P}_j^{(\text {jam})}, \end{aligned}$$

biasing the noise estimate $Z_{c_0}$ and thus the adaptive threshold $\lambda _{c_0} = \alpha Z_{c_0}$. For mean-level CA-CFAR, this gives

17 $$\begin{aligned} Z_{\text {CA}} = \frac{1}{|\mathcal {R}(c_0)|} \sum _{j \in \mathcal {R}(c_0)} \bar{P}_j, \qquad |\mathcal {R}(c_0)| = 2N_r. \end{aligned}$$

The design goal is to create distributed intersections with $\mathcal {R}(c_0)$ and the CUT over typical sensing intervals, preventing adaptive estimators from reliably identifying persistently clean reference subsets. On PU-absent channels this drives $\bar{P}_{c_0}> \lambda _{c_0}$ frequently, sustaining the illusion of occupancy, while on PU-present channels the PU energy in the CUT remains sufficiently dominant for reliable detection. The following subsection evaluates this enforcement balance across the full hybrid suite.

### Performance evaluation of controlled interference

#### Design objectives and optimization framework

The comb-sweep counter-access strategy must balance two enforcement objectives: inducing high false-alarm probability on PU-absent channels (deceptive occupancy, denying unauthorized access) while maintaining high detection probability on PU-present channels (protecting incumbents). Unlike conventional CFAR operation—where $P_{\mathrm{fa}}$ and $P_d$ are coupled through threshold scaling in a fixed environment—this work exploits *environmental manipulation*: structured, window-aware interference alters the statistics driving the SU’s reference-based estimate $Z_{c_0}$ and thus its adaptive decision threshold. Selecting the JSR requires systematic evaluation: insufficient jamming permits intermittent clean references that allow opportunistic access, while excessive jamming may over-inflate thresholds for certain hybrids and degrade PU detection.

We explore a two-dimensional operating space $(\mathrm{JSR},\beta )$ to identify robust enforcement regimes. JSR controls the physical interference level, affecting both detection and denial. The parameter $\beta \in [0,1]$ is an *administrator-side* weighting that reflects enforcement priorities: *β* near 1 prioritizes PU protection (ensuring incumbents are never missed), while *β* near 0 prioritizes access denial (maximizing false occupancy on vacant channels). These parameters are interdependent—the utility of a given JSR depends on enforcement emphasis, and vice versa.

To quantify this trade-off, we define performance metrics that reward both high central tendency and consistency across SNR. For each detector variant *i* and metric $P_x \in \{P_d^{(\mathrm{PU})},\,P_{\mathrm{fa}}^{(\mathrm{vacant})}\}$, we compute

18 $$\begin{aligned} M_{P_x,i} = \operatorname {median}(P_{x,i})\;\Big (1-\operatorname {IQR}(P_{x,i})\Big ), \end{aligned}$$

where $P_{x,i}$ is the probability vector over SNR *∈ [-5,25]* dB. The median captures central performance while $(1-\operatorname {IQR})$ penalizes variability, with $\operatorname {IQR}=Q_3-Q_1$ the interquartile range. Since probabilities lie in [0, 1], the factor $(1-\operatorname {IQR})$ approaches zero for erratic performance and unity for perfectly stable performance. This construction is preferred over simple averaging because it robustly handles outliers while explicitly valuing consistency.

The optimization objective combines detection and denial across all *N* evaluated hybrids (*N=13*), weighted by the administrator’s priority parameter *β*:

19 $$\begin{aligned} \zeta (\mathrm{JSR}, \beta )&= \beta \ln \left( \sum _{i=1}^{N} M_{P_{d}^{(\mathrm{PU})},i}\right) \nonumber \\&\quad + (1 - \beta ) \ln \left( \sum _{i=1}^{N} M_{P_{\mathrm{fa}}^{(\mathrm{vacant})},i}\right) \end{aligned}$$

The logarithmic transformation prevents either term from dominating by magnitude alone, effectively penalizing catastrophic failure in either objective. High *β* (e.g., 0.9) weights the first term heavily, favoring configurations that guarantee PU detection even if denial is incomplete. Low *β* (e.g., 0.1) weights the second term heavily, favoring configurations that maximize denial even if some conservative detectors occasionally miss PUs at extreme SNR.

We perform a grid search over $\mathrm{JSR}\in [-10,-1]$ dB (1 dB steps) and $\beta \in [0.1,0.9]$ (0.1 steps), yielding 90 operating points. Each is evaluated via Monte Carlo simulation with 10,000 iterations under the assumptions in “4.1” section. Crucially, *β* is an administrator design choice, not an SU parameter–secondary receivers use fixed CFAR scaling $\lambda _{c_0}=\alpha Z_{c_0}$ designed for nominal interference-free operation.

#### Full-suite analysis: bifurcation and hybrid-specific sensitivities

**Fig. 7:**
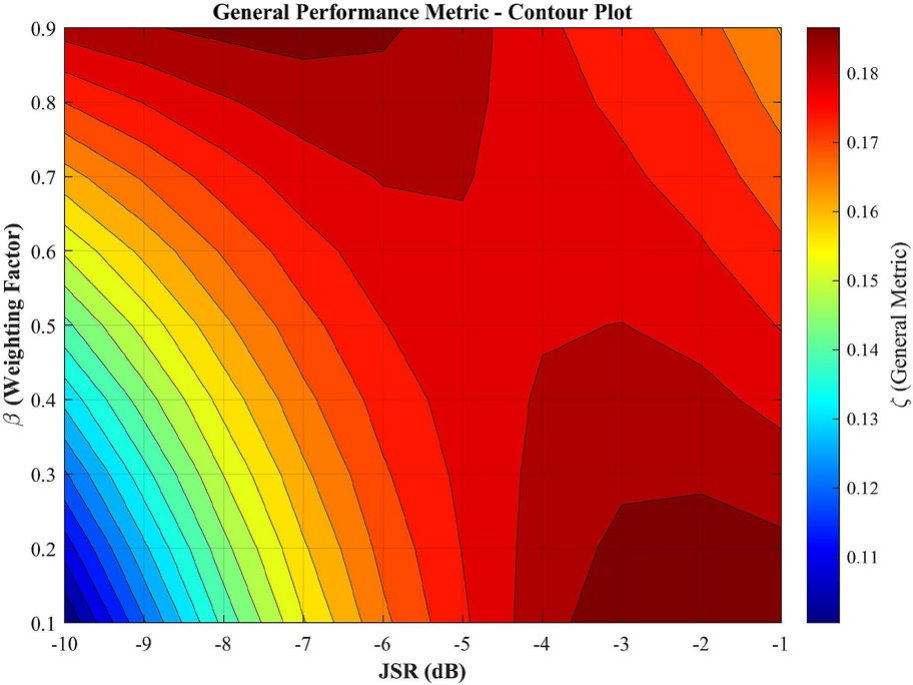
Optimization landscape over $(\mathrm{JSR},\beta )$ for the full hybrid suite, including OSSO-CFAR, OSGO-CFAR, and WCA-CFAR. The primary denial-optimal region near JSR *≈ -2* dB at low *β* contrasts with a secondary protection-oriented region at lower JSR and high *β*.

**Fig. 8:**
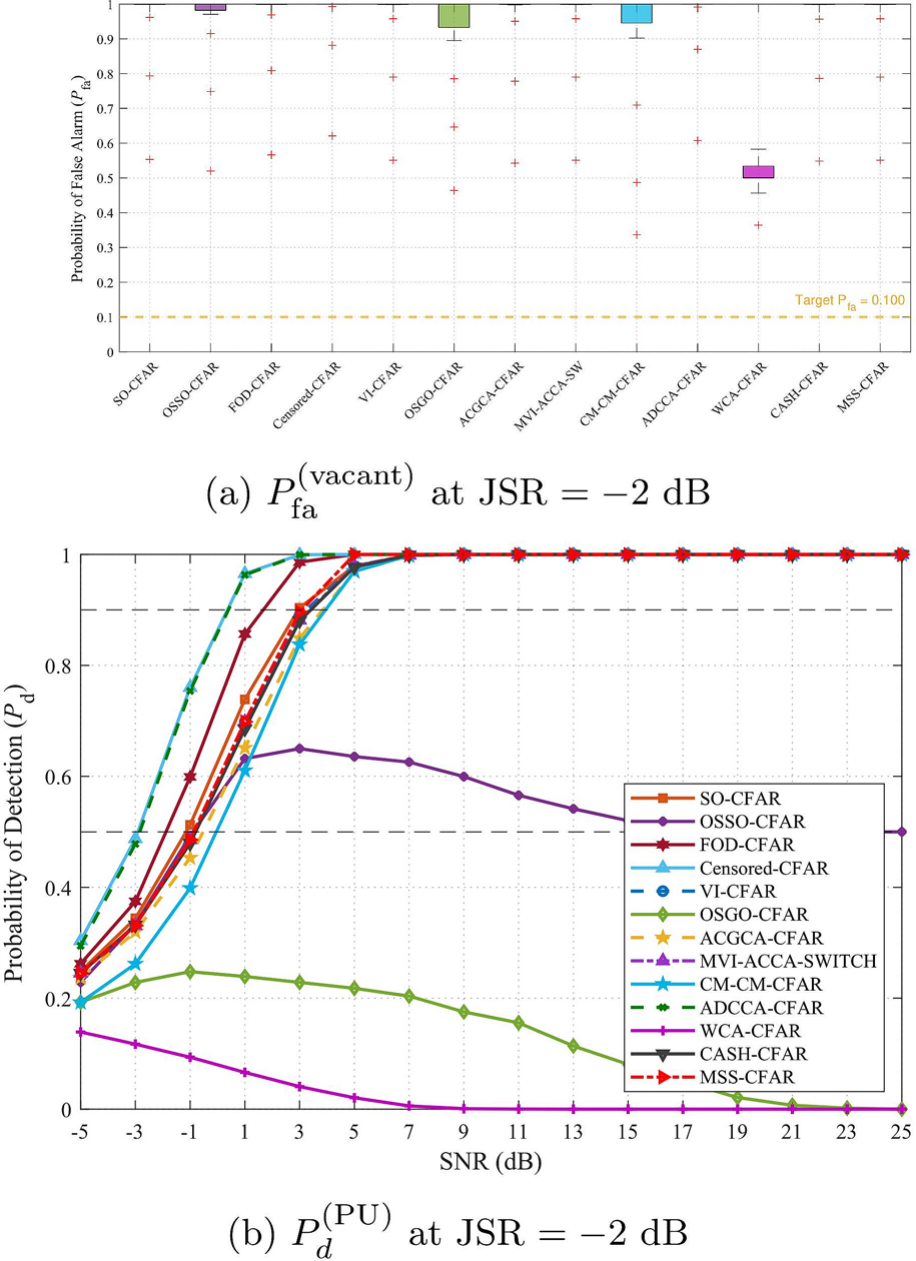
Performance at the global optimum (JSR *= -2* dB, *β =0.1*) showing deceptive occupancy on vacant channels and preserved PU detection under comb-sweep counter-access.

Figure 7 presents the optimization landscape for the full hybrid suite, including OSSO-CFAR, OSGO-CFAR, and WCA-CFAR. The contour reveals a bifurcated structure with two distinct operating regions. The primary region at JSR *≈ -3* to *-1* dB and low $\beta \approx 0.1$–0.2 exhibits the global maximum at (JSR *= -2* dB, *β =0.1*), achieving comprehensive denial across most hybrids. Figure 8 illustrates performance at this optimum: deceptive occupancy approaches unity for mean-level hybrids (panel a), while $P_d^{(\mathrm{PU})}$ remains high (panel b). A secondary region emerges at lower JSR *≈ -9* to *-5* dB with high $\beta \approx 0.8$–0.9, prioritizing PU detection uniformity over complete denial.

This bifurcation arises because fusion- and weighting-based hybrids (OSSO, OSGO, WCA) respond differently to comb-sweep contamination. OSSO-CFAR can partially evade deceptive occupancy at moderate JSR by selecting the less-contaminated reference half through smallest-of fusion, reducing $P_{\mathrm{fa}}^{(\mathrm{vacant})}$ and weakening denial. Conversely, OSGO-CFAR and WCA-CFAR are inherently conservative: persistent contamination on either side (OSGO) or homogeneity-driven weight fluctuations (WCA) inflate $Z_{c_0}$ and the adaptive threshold. At higher JSR, this inflation can degrade $P_{d}^{(\mathrm{PU})}$ when the reference-driven threshold rises faster than the PU-dominated CUT statistic.

The metric $\zeta (\mathrm{JSR},\beta )$ thus identifies two enforcement philosophies: (i) a *denial-dominant* regime (low *β*, high JSR) maximizing deceptive occupancy across the suite, and (ii) a *protection-dominant* regime (high *β*, low JSR) sacrificing complete denial to maintain stable PU detection for conservative variants. For an unauthorized SU, these hybrids introduce strategic uncertainty: OSSO may reduce denial at moderate JSR, while OSGO and WCA may suffer detection degradation at high JSR. Because the SU cannot predict the administrator’s chosen JSR and comb-sweep intersections vary across time and location–these hybrids cannot guarantee simultaneously low $P_{\mathrm{fa}}^{(\mathrm{vacant})}$ and high $P_{d}^{(\mathrm{PU})}$. This manifests the security paradox: resilience mechanisms that help under natural interference create new failure modes under algorithm-aware counter-access.

#### Reduced-suite analysis: unified denial plateau

**Fig. 9:**
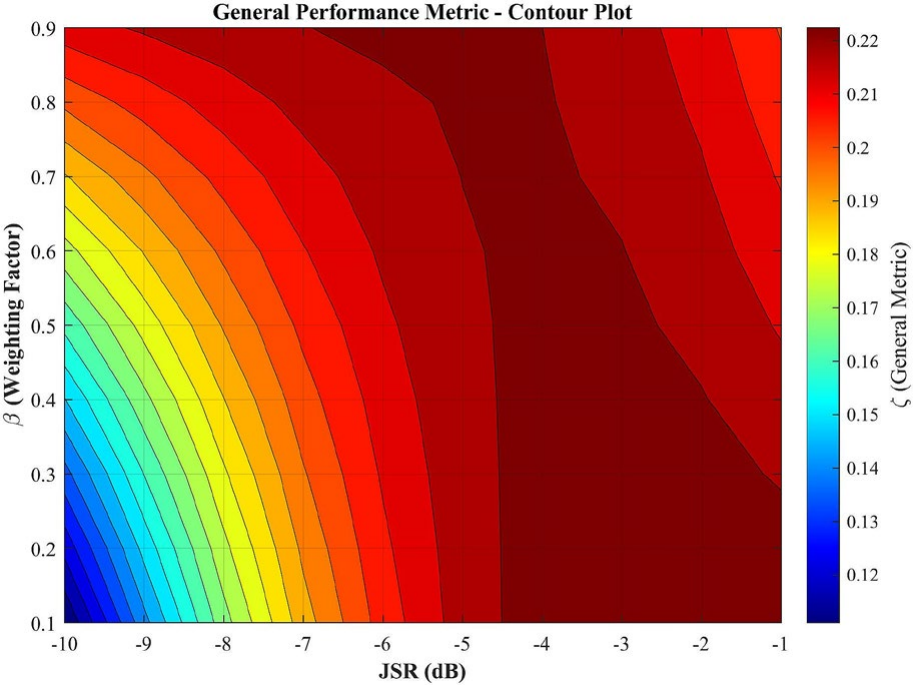
Optimization landscape excluding OSSO-CFAR, OSGO-CFAR, and WCA-CFAR, revealing a unified optimal region at JSR *∈ [-5,-1]* dB with consistent performance across $\beta \in [0.1,0.6]$.

If the adversary is unlikely to use OSSO-CFAR, OSGO-CFAR, or WCA-CFAR—or if the administrator can neutralize these variants through other means—then excluding them simplifies enforcement. Figure 9 shows the optimization landscape for the reduced suite. The bifurcated structure collapses into a single, unified optimal region spanning JSR *∈ [-5,-1]* dB with consistent peak performance across $\beta \in [0.1,0.6]$.

In this broad plateau, all remaining hybrids (the majority of our suite) exhibit homogeneous behavior: near-unity false alarms on vacant channels (sustained deceptive occupancy) while maintaining high PU detection. This robustness eliminates the need for precise power tuning—any JSR within *[-5,-1]* dB yields effective denial while preserving PU protection.

This consolidation offers three operational advantages. *First*, eliminating competing optima removes strategic ambiguity, giving the administrator a clear design space. *Second*, the 4 dB JSR plateau provides robustness against channel uncertainty; performance remains nearly invariant across this span, critical because precise JSR at the SU cannot be guaranteed under path loss variability, fading, and unknown implementation losses. *Third*, the reduced suite exhibits predictable denial behavior: systematic reference contamination combined with intermittent CUT intersections drives deceptive occupancy toward unity across the plateau, ensuring effective denial regardless of which specific non-fusion/weighting hybrid the unauthorized SU employs.

#### Validation and robustness assessment

**Fig. 10:**
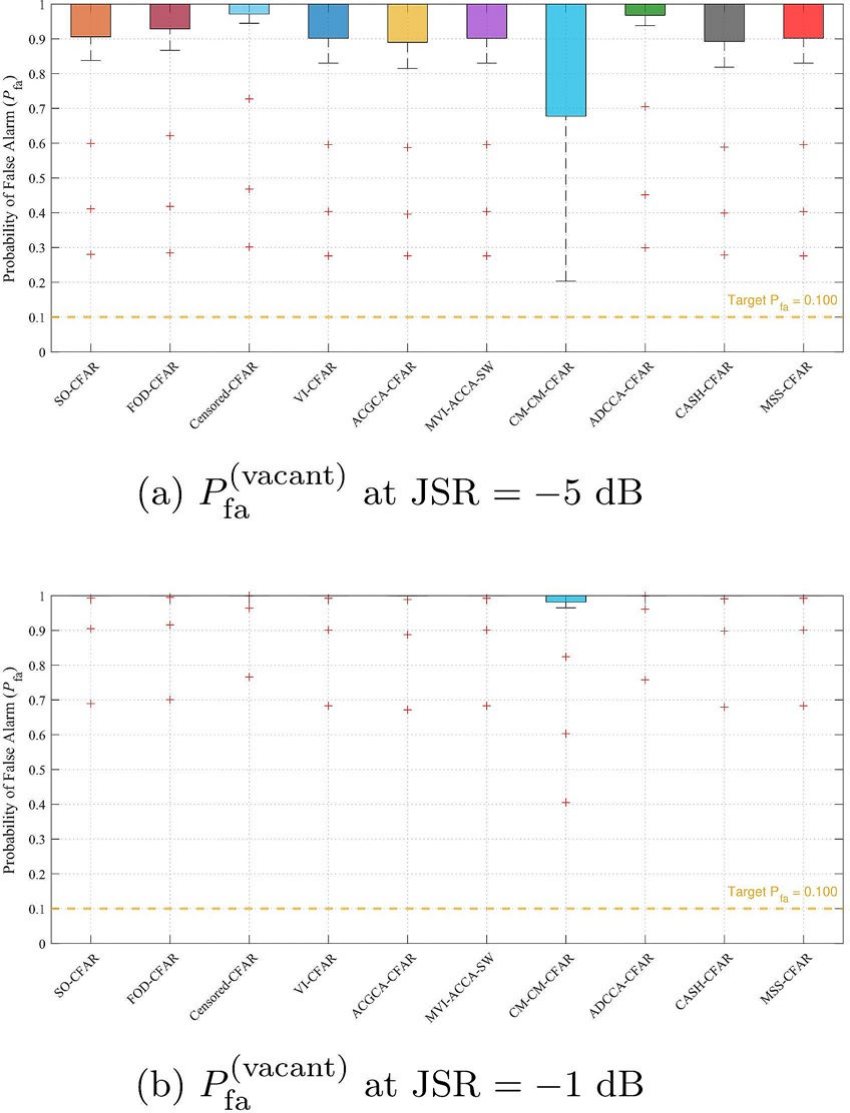
False-occupancy comparison at denial-plateau boundaries (OSSO/OSGO/WCA excluded): the reduced hybrid suite maintains consistently high $P_{\mathrm{fa}}^{(\mathrm{vacant})}$ across the 4 dB JSR span.

To validate plateau robustness, we compare performance at the optimal region boundaries: JSR *= -5* dB and JSR *= -1* dB (OSSO/OSGO/WCA excluded). Figure 10 demonstrates that deceptive-occupancy induction remains uniformly effective across both JSR values. At JSR *= -5* dB (panel a), $P_{\mathrm{fa}}^{(\mathrm{vacant})}$ concentrates near unity, indicating consistent denial across SNR. At JSR *= -1* dB (panel b), the same high-denial behavior persists despite the 4 dB power increase (equivalent to six-fold jamming power elevation).

**Fig. 11:**
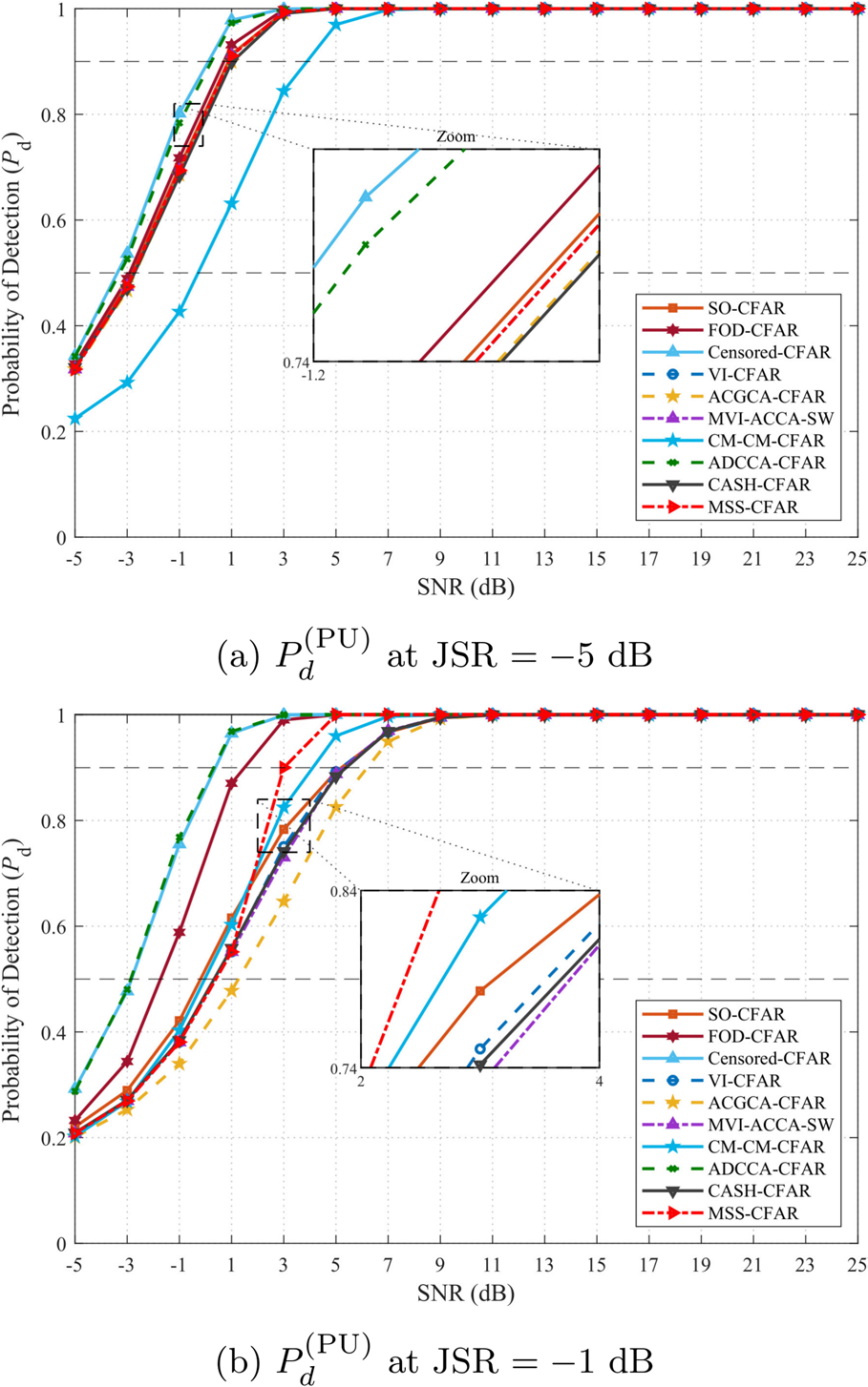
Detection comparison at denial-plateau boundaries (OSSO/OSGO/WCA excluded): nearly overlapping $P_{d}^{(\mathrm{PU})}$ curves demonstrate preserved PU protection across the 4 dB JSR span.

Figure 11 shows that PU detection remains similarly stable. At JSR *= -5* dB (panel a), the reduced-suite hybrids achieve rapid transition to high $P_{d}^{(\mathrm{PU})}$ as SNR increases, reaching near-unity detection within the operational range. At JSR *= -1* dB (panel b), detection curves closely track those at *-5* dB, indicating that the six-fold power increase does not materially degrade PU detectability; PU energy in the CUT remains sufficiently dominant relative to the reference-driven threshold.

The edge-case validation confirms that the optimal region functions as a true denial plateau. An administrator can select any JSR value within *[-5,-1]* dB and achieve near-identical enforcement outcomes: sustained deceptive occupancy on PU-absent channels while maintaining reliable PU detection. This 4 dB tolerance accommodates real-world propagation uncertainties, including path loss variability (*± 2* dB typical for land mobile radio), shadowing effects, and unknown SU receiver implementation losses.

In summary, the comb-sweep interference strategy reliably forces advanced CFAR-equipped SUs to falsely perceive vacant channels as occupied (achieving nearly 100% $P_{\mathrm{fa}}^{(\mathrm{vacant})}$) across a practical JSR range, while legitimate PU signals continue to be detected with high probability. This dual-objective capability—denying unauthorized access while preserving spectrum safety—validates the algorithm-aware countermeasure approach for administrative spectrum enforcement in contested cognitive radio environments.

### Integration with P25 protocol timing

The comb-sweep cycle ($T_{\text {cycle}} \approx 1.6$ ms) and period ($T_{\text {period}} \approx 416~\mu$s) are designed to align with APCO Project 25 (P25) waveform timing^45^. The sweep period matches the SU’s FFT-based sensing interval, ensuring successive snapshots encounter statistically different interference realizations rather than quasi-static jammer states. This synchronization prevents unauthorized SUs from identifying repeatable interference-free windows for opportunistic access, while operating at sub-millisecond cadence makes the interference indistinguishable from legitimate fast state changes in trunked public-safety systems^46^.

By matching the sweep period to the SU sensing interval, each snapshot encounters reference contamination, CUT-neighborhood intersections, or both, precluding stable occupancy modeling. The administrator’s protocol-level knowledge (channelization, signaling structure, timing constraints) creates asymmetric information advantage: the jammer appears as plausible network dynamics rather than persistent anomaly^47^, violating the environmental stationarity assumptions underlying adaptive CFAR logic.

This synchronization strategy assumes the unauthorized SU senses using P25-compatible timing parameters (FFT size, sampling rate, and hence sensing interval $T_0$). This is not merely a convenient assumption: because the SU’s own cell-power statistic $\bar{P}_c$ (Eq. (7)) is formed by aggregating $M = \lceil B_{\mathrm{PU}}/B_{\mathrm{FFT}} \rceil$ FFT bins matched to the known, fixed P25 channel bandwidth $B_{\mathrm{ch}} = 12.5$ kHz, an SU that deviates from standard timing parameters mismatches its own cell-to-bandwidth mapping, degrading its own PU-detection accuracy. Deliberate deviation is therefore self-defeating for an SU whose primary objective is reliable spectrum sensing, and standard P25 timing is consequently the SU’s rational operating point rather than an assumption imposed by the administrator. Moreover, irrespective of the specific FFT size or sampling rate an SU adopts, any spectrally meaningful implementation must ultimately resolve its bins back to the same fixed channel grid *c = 1,… ,C* defined by $B_{\mathrm{ch}}$, since this grid is dictated by the physical P25 channelization rather than by the SU’s internal FFT configuration. The comb-sweep jammer targets this channel grid directly (“Signal model and deceptive occupancy framework” section), so its spectral alignment with the SU’s reference window is preserved regardless of the SU’s chosen FFT parameters; only the SU’s *temporal* sensing cadence ($T_0$) is affected by such variation, weakening the precise period-matching advantage described above without disrupting the underlying channel-level contamination. We note further that the comb-sweep countermeasure’s denial effectiveness in “Performance evaluation of controlled interference” section stems from the statistical power balance between jammer and reference-window occupancy rather than from exact timing knowledge alone, and our companion classical-CFAR study^14^ demonstrated that this denial mechanism is similarly insensitive to administrator uncertainty over the SU’s CFAR window geometry ($N_r \in \{6,\ldots ,20\}$, $N_g \in \{0,1,2\}$), with negligible performance variation across all tested configurations. Formally extending this robustness analysis to unintentional SU timing-cadence deviations is identified as future work in “Conclusion and future work” section.

In summary, comb-sweep jamming combines window-aware interference with protocol-layer timing synchronization to reassert administrative control via structured, sparse spectral excitation rather than indiscriminate wideband flooding, thereby preserving PU communications^45,46^.

## Conclusion and future work

This paper evaluated hybrid frequency-domain CFAR architectures for cognitive radio spectrum sensing under heterogeneous interference and structured jamming. Among the 13 variants, FOD-CFAR demonstrated superior robustness under heavy jamming through global ordering and jump detection, followed by SO-CFAR and OSSO-CFAR –the latter proving effective provided that at least one side of the reference window remains predominantly clear. However, this resilience creates a security paradox: adaptive sensing that protects honest SUs can also enable noncompliant SUs to evade spectrum denial. The proposed comb-sweep jamming countermeasure addresses this dual-use vulnerability by exploiting CFAR reference-window dependencies. By systematically contaminating reference cells, it induces near-universal false alarms on vacant channels (denial plateau at JSR *∈ [-5, -1]* dB) while preserving PU detection probability, demonstrating that algorithm-aware interference can restore administrative control against advanced adaptive receivers. Future work will focus on FPGA-based deployment of FOD-CFAR and over-the-air validation. Key challenges in this transition include timing jitter perturbing the synchronization between the comb-sweep period and the SU’s FFT sensing interval, non-ideal propagation effects (multipath fading, shadowing, Doppler spread) introducing non-stationarity in reference-cell statistics beyond AWGN assumptions, imperfect synchronization with P25 trunked network timing, the pipeline design requirements of real-time FOD-CFAR sorting within the 416 *μ*s sensing interval, and quantifying comb-sweep enforcement effectiveness under unintentional SU timing-cadence deviations (e.g., non-standard FFT size or sampling rate), extending the window-geometry robustness analysis of^14^ to this timing dimension.

## Data Availability

All data generated or analysed during this study are included in this published article. The findings are based entirely on Monte Carlo simulations, with all parameters and configurations fully described within the manuscript, making the results independently reproducible.
